# Impact of general anaesthesia on breast cancer survival: a 5-year follow up of a pragmatic, randomised, controlled trial, the CAN-study, comparing propofol and sevoflurane

**DOI:** 10.1016/j.eclinm.2023.102037

**Published:** 2023-06-09

**Authors:** Mats Enlund, Anders Berglund, Anna Enlund, Johan Lundberg, Fredrik Wärnberg, Dong-Xin Wang, Andreas Ekman, Rebecca Ahlstrand, Per Flisberg, Lars Hedlund, Ingrid Östlund, Leif Bergkvist, CAN-study group

**Affiliations:** a Centre for Clinical Research, Västmanland Hospital Västerås, University of Uppsala, Sweden; b Epistat AB, Uppsala, Sweden; c Department of Perioperative and Intensive Care, Skåne University Hospital, Lund, Sweden; d Department of Surgical Sciences, Uppsala University, Uppsala, Sweden; e Department of Surgery, Sahlgrenska University Hospital, Gothenburg, Sweden; f Institute of Clinical Sciences, Sahlgrenska Academy at University of Gothenburg, Sweden; g Department of Anaesthesiology, Peking University First Hospital, Beijing, China; h Department of Anaesthesia and Intensive Care, Kalmar Hospital, Kalmar, Sweden; i Department of Anaesthesia and Intensive Care, Örebro University Hospital, Örebro, Sweden; j Department of Anaesthesia and Intensive Care, Helsingborg Hospital, Helsingborg, Sweden; k Department of Anaesthesia and Intensive Care, Skellefteå Hospital, Sweden

**Keywords:** Breast cancer, Anaesthesia, Inhalation, Intravenous, Survival analysis

## Abstract

**Background:**

Anaesthesia may impact long-term cancer survival. In the Cancer and Anaesthesia study, we hypothesised that the hypnotic drug propofol will have an advantage of at least five percentage points in five-year survival over the inhalational anaesthetic sevoflurane for breast cancer surgery.

**Methods:**

From 2118 eligible breast cancer patients scheduled for primary curable, invasive breast cancer surgery, 1764 were recruited after ethical approval and individual informed consent to this open label, single-blind, randomised trial at four county- and three university hospitals in Sweden and one Chinese university hospital. Of surveyed patients, 354 were excluded, mainly due to refusal to participate. Patients were randomised by computer at the monitoring organisation to general anaesthesia maintenance with either intravenous propofol or inhaled sevoflurane in a 1:1 ratio in permuted blocks. Data related to anaesthesia, surgery, oncology, and demographics were registered. The primary endpoint was five-year overall survival. Data are presented as Kaplan-Meier survival curves and Hazard Ratios based on Cox univariable regression analyses by both intention-to-treat and per-protocol. EudraCT, 2013-002380-25 and ClinicalTrials.gov, NCT01975064.

**Findings:**

Of 1764 patients, included from December 3, 2013, to September 29, 2017, 1670 remained for analysis. The numbers who survived at least five years were 773/841 (91.9% (95% CI 90.1–93.8)) in the propofol group and 764/829 (92.2% (90.3–94.0)) in the sevoflurane group, (HR 1.03 (0.73–1.44); P = 0.875); the corresponding results in the per-protocol-analysis were: 733/798 (91.9% (90.0–93.8)) and 653/710 (92.0% (90.0–94.0)) (HR = 1.01 (0.71–1.44); P = 0.955). Survival after a median follow-up of 76.7 months did not indicate any difference between the groups (HR 0.97, 0.72–1.29; P = 0.829, log rank test).

**Interpretation:**

No difference in overall survival was found between general anaesthesia with propofol or sevoflurane for breast cancer surgery.

**Funding:**

10.13039/501100004359Swedish Research Council; 10.13039/100019032Uppsala-Örebro Regional Research Council; Västmanland Regional Research Fund; Västmanland Cancer Foundation; Stig and Ragna Gohrton Foundation; Birgit and Henry Knutsson Foundation.

**Research in context**

**Evidence before this study**

A possible relationship between long-term survival and the choice of anaesthetic agent for maintenance of anaesthesia for cancer surgery is based on biochemical, immunological, and animal studies along with observational studies. The inflammatory response initiated by the surgical trauma is maintained and increased by volatile anaesthetics but attenuated by propofol. The hypothesis that propofol may be beneficial for maintenance of anaesthesia has been questioned for breast cancer in several retrospective studies.

**Added value of this study**

In this randomised controlled trial, involving radical curative removal of primary breast cancer, patients were given propofol or sevoflurane for maintenance of general anaesthesia. The study question was whether propofol increases long-term over-all survival, compared with sevoflurane.

**Implications of all the available evidence**

The result implies that long-term survival for breast cancer is unaffected by the choice of anaesthetic for maintenance of general anaesthesia. Equivalence is difficult to prove due to the difficulty of attaining sufficient statistical power for the relatively rare outcome of mortality, even with large sample sizes. Furthermore, since Evidence-based medicine requires at least two well-conducted randomised clinical trials to influence a guideline, further trials are needed.

## Introduction

With more than 700,000 deaths annually, breast cancer is the fourth most common cause of death for both sexes from cancer globally.^1^ Surgical excision is a cornerstone of treatment and anaesthesia is a prerequisite for this.

Animal-, in vitro-, and retrospective clinical studies demonstrate that survival may be affected by the anaesthetic technique for many solid cancers, e.g., gastroesophageal cancer and hepatobiliary cancer.^2^^,^^3^ Plausible biological explanations exist, e.g., opposite effects on the immune system with suppression from inhaled anaesthetics, genotoxicity from this group, and also upregulation of hypoxia-inducible factor, making cancer cells more viable, while propofol seems to have the opposite effect.^4^^,^^5^ For breast cancer, vascular endothelial growth factor C, which promotes angiogenesis and metastasis, is increased under sevoflurane/opioid anaesthesia, but not under propofol/paravertebral anaesthesia.^6^ In addition, the antitumor activity of natural killer cells was higher in women anaesthetised with propofol/paravertebral for breast cancer compared with women anaesthetised with sevoflurane/opioid, and the same relationship was true for cancer cell apoptosis.^7^^,^^8^ It seems that intravenous propofol is a better choice than inhaled anaesthetics. However, despite these data, subsequent retrospective studies suggest that the hypothesis does not apply to breast cancer.^9^, ^10^, ^11^, ^12^, ^13^ Moreover, in a prospective RCT, Sessler et al. found comparable rates of breast cancer recurrence in a comparison of a combination of propofol and paravertebral block with sevoflurane and opioids.^14^

We initiated the Cancer and Anaesthesia study (CAN), based on survival outcomes from an early retrospective study, involving both breast cancer and colorectal cancer.^15^ The aim was to prospectively include patients for surgery of these cancer types and randomise to general anaesthesia based on propofol or sevoflurane and then compare long-term survival in the CAN study.^16^ In this report, we present the data for the breast cancer patients, as the inclusions to the colorectal part were completed only recently. Five-year overall survival was decided as the primary outcome. We hypothesised that propofol-based general anaesthesia improves five-year survival for breast cancer by five percentage points.

## Methods

This pragmatic, randomised, open label, single-blinded, multicentre, and binational study was approved by the Regional Ethics Committee in Uppsala, Sweden, Dnr 2013/314, on August 14, 2013 (Email: registrator@etikprovning.se), registered at the European Clinical Trials Database, EudraCT, on August 16, 2013 (2013-002380-25), and at ClinicalTrials.gov on November 2, 2013 (NCT01975064). A separate approval was obtained in Beijing, China (Peking University First Hospital, 2015/1003). Four county hospitals and three university hospitals in Sweden and one university hospital in China participated. The study protocol can be found at https://regionvastmanland.se/globalassets/vardgivare-och-samarbetspartners/forskning-och-utbildning/forskning/protokoll/c_679870-l_3-k_protocol-can-version-28-oct-2016.pdf.

Patient screening was organised slightly differently at the participating sites, usually performed at review of the surgical program or before pre-anaesthesia visits. All patients were included by the local primary investigator or another listed anaesthetist or surgeon after written and verbal information and signed consent.

Inclusion criteria: The patients should be at least 18 years of age, both sexes, scheduled for elective, curable, invasive primary breast cancer surgery with general anaesthesia with or without local infiltration or regional anaesthesia with a local anaesthetic agent.

Exclusion criteria: Patients scheduled for emergency surgery, palliative surgery, in-situ cancer, presence of any contraindication (according to the substances’ valid Summary of Product Characteristics), or lack of suitability for any reason, as judged by the local investigator, e.g., communicative disturbances (language or intellectual) were not included in the study.

### Randomisation and masking

The study patients were randomly allocated to anaesthesia maintenance with either propofol or sevoflurane in a 1:1 ratio. The computerised randomisation list was generated centrally by the monitoring organisation, Uppsala Clinical Research Center (UCR), in a permuted block fashion and transferred to a sequence of sealed, opaque, consecutively numbered envelopes with unique numbers for each study site. The envelopes were distributed to each study site at its initiation meeting by the Coordinating-PI. When a patient was considered eligible for the study and gave informed written and verbal consent, the randomisation was performed by opening the next envelope in sequence. Once assigned to a treatment group by randomisation, a patient could not be withdrawn from the study population except on the patient's own initiative. Furthermore, cross-over between treatment assignments (propofol to sevoflurane, or vice versa) was not permitted. Blinding did not apply to participating anaesthetists, but to patients, statisticians, and those assessing outcomes.

### Procedures

The propofol group received propofol for both induction and maintenance of general anaesthesia. The sevoflurane group received propofol for induction and sevoflurane for maintenance of general anaesthesia. There were no protocol-specific restrictions for concurrent medication in this pragmatic study. In both groups, adjuvant drugs, such as opioids and neuromuscular blocking agents were administered according to standard institutional procedures at each site, as was postoperative analgesia. General anaesthesia was maintained according to randomisation and otherwise performed according to local practice, including the addition of regional anaesthesia. However, the use of nitrous oxide was not allowed. Perioperative opioid consumption was recorded for both intravenous and oral opioids. The use of perioperative local infiltration or a regional nerve block with a local anaesthetic agent was recorded.

Data including details of general anaesthesia, American Society of Anesthesiologists (ASA) classification, drug use, surgical procedure (breast conservation or mastectomy), demographics, smoking, alcohol use, and general health were recorded in an electronic case report form (eCRF) (specified and listed in Appendix 1). Predefined complications were summarised at a 30-day follow-up of patient records. Tumour specific variables, such as cancer stage (tumour size and lymph node pathology), any metastasis, and different prognostic markers (receptor status, cell proliferation, human epidermal growth factor receptor 2-status (HER2)) were extracted by record-linkage, combining the unique individual Swedish social security number with the Swedish National Breast Cancer Quality Register, at follow-up, as was vital status. For the patients in Beijing, a translated copy of the Swedish National Breast Cancer Quality Register was constructed in a separate internet-based program to capture the same data collected from telephone calls with patients or their relatives.

In accordance with the principles of Good Clinical Practice (GCP), monitoring of the study was arranged by the Sponsor (Uppsala University, Centre for Clinical Research, Västerås, Sweden). UCR was appointed to monitor this study for the Swedish sites and for the central monitoring of the database. For the Beijing site, the Sponsor appointed a local monitoring organisation, Peking University Clinical Research Institute, to perform on-site monitoring activities and regular contacts. The independent data management of the clinical database was managed by UCR, based on a specific Data Management Plan (DMP). The eCRF served as the clinical database for the study. In addition, UCR was responsible for set-up, support, and management of the eCRF. All demographic, anaesthesia related, and surgery related data were collected in the database (Appendix 1). Data management also included handling of queries to resolve any inconsistencies detected by the quality control procedures. Subsequently, eCRF data were subject to both logical computerised checks and manual validation checks against listings in accordance with the study specific DMP. All inconsistencies detected during these procedures were resolved through queries, being issued to the monitor and investigational site personnel.

A preceding or subsequent anaesthetic in temporal proximity to the index procedure was considered an effect modifier necessary to identify. An executive decision was made to define the time interval to ±1 year from the index operation. Patients anaesthetised within this time frame before the index operation were identified during the pre-anaesthesia interview, and those anaesthetised after the index operation were sought in the electronic patient record system at the 1-year follow-up. It was noted in each patient's electronic medical record that, if possible, during the first postoperative year, the same anaesthetic agent as during the index procedure should be used, if the need for general anaesthesia should arise. Complications, appearing up to 30 days post-surgery, were collected in the eCRF.

### Outcomes

All outcome data was sent to UCR. The 5-year overall survival was the primary outcome. The 1-year survival was the secondary outcome, although of small interest for breast cancer with its high survival rate. The 1-year survival has been analysed and presented earlier.^17^

### Statistical analysis

We hypothesised, that propofol-based general anaesthesia would improve outcome by five percentage-points compared with sevoflurane. This difference was found in our first retrospective study (breast- and colo-rectal cancer), albeit the P-value for the Hazard Ratio for sevoflurane vs propofol was 0.051 after adjustments for uneven distribution of confounders and effect modifiers.^15^ We calculated the statistical power from the retrospective study and national Swedish breast cancer survival data.^15^^,^^16^ With 1650 patients in the breast cancer cohort, 80% power was obtained to detect a survival difference of five percentage points with a P-value of less than 0.05. Adding an additional 114 patients (7%) provided a reasonable safety margin for patient loss or technical failures.

The Intention-to-Treat (ITT) population included all eligible breast cancer patients between December 3, 2013, and September 29, 2017, that were not excluded due to exclusion criteria or lost to follow up. The per-protocol (PP) population included only the patients from the ITT population who received the allocated intervention in each arm, respectively.

Demographic and postoperative data were evaluated by descriptive methods, and all variables are presented as aggregated data. Categorical variables are summarised in frequency tables (presenting frequencies and proportions) by type of anaesthesia. The quantitative variables are summarised by number of observations, median, inter-quartile ranges (IQR) by anaesthesia. A separate summary is presented for patients undergoing one or more procedures in general anaesthesia in addition to the study procedure, as well as pooled summaries. Overall survival is presented as Kaplan-Meier curves and hazard ratios based on univariable Cox regression analyses for overall deaths. In addition, univariable Cox regression models were estimated for pre-specified subgroups (see Table S1 and Figs. S3–S6), but no formal testing for effect modifications was performed. Overall survival was defined from index date to death due to any causes or end of follow up. In addition, for the Swedish subjects, cause-specific survival was calculated as the time from index date to death due to breast cancer, in where death due to other causes than breast cancer was considered as censored observations. A P-value ≤0.05 was considered statistically significant and the hypothesis testing was 2-sided. The statistical analysis was carried out using R version 4.2.1. (R Foundation for Statistical Computing, Wirtschaftsuniversität Wien, Vienna, Austria).

### Role of the funding source

The funders of the study had no role in study design, data collection, data analysis, data interpretation, writing of the report, or the decision to submit for publication.

## Results

**Fig. 1:**
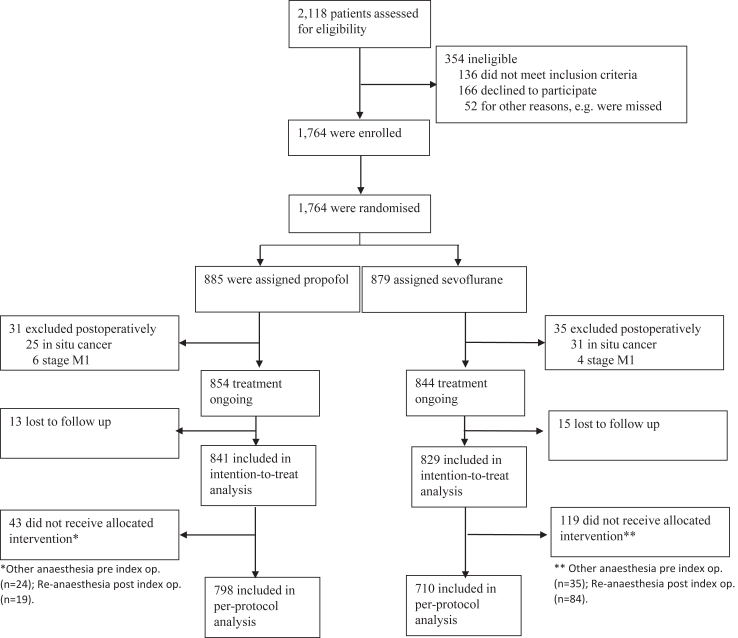
Flow diagram for the CAN-study randomising between propofol and sevoflurane during breast cancer surgery.

The predefined patient sample was obtained between December 3, 2013, and September 29, 2017. From 2118 eligible breast cancer patients, 1764 were recruited for the study (number from the respective site in Appendix 2). Eventually, 1670 remained for ITT, 841 in the propofol group and 829 in the sevoflurane group. In all, 162 patients received the non-intended drug, either during the year before the index operation, or for a re-operation within the first postoperative year, rendering in 1508 patients for PP-analysis, 798 in the propofol group and 710 in the sevoflurane group (Fig. 1). The data extraction was performed on November 1, 2022. Patient follow-up time was 76.7 [65.0, 86.6] and 76.9 [65.0, 87.0] months (median ± IQR) for the propofol and sevoflurane groups, respectively.

Demographics and clinical data are presented in Table 1. The two groups were comparable in baseline characteristics except for triple negative hormone receptor status, 41 patients in the propofol group (6.6%) vs 26 patients in the sevoflurane group (4.3%) with 26.1% missing values in the overall patient cohort. Regarding anaesthesia associated variables, differences were noted in the use of intraoperative parenteral opioids (Table 2). Remifentanil was used more in conjunction with propofol (86.3% of patients vs 42.1% in the sevoflurane group), while fentanyl was used more in combination with sevoflurane (69.5% vs 24.4% in the propofol group).

**Table 1:** Demographics and clinical characteristics of the study cohort.

	Propofol	Sevoflurane	Total	Missing
	n (%)	n (%)	n (%)	%
**All subjects**	841 (100.0)	829 (100.0)	1670 (100.0)	0.0
**Sex**				0.0
Female	836 (99.4)	823 (99.3)	1659 (99.3)	
Male	5 (0.6)	6 (0.7)	11 (0.7)	
**Age, median [IQR]**	64.0 [52.0, 71.0]	63.0 [53.0, 71.0]	64.0 [52.0, 71.0]	0.0
**BMI, median [IQR]**	27.0 [24.0, 30.0]	27.0 [24.0, 30.0]	27.0 [24.0, 30.0]	1.6
**Follow up in months, median [IQR]**	76.7 [65.0, 86.6]	76.9 [65.0, 87.0]	76.5 [65.0, 86.0]	0.0
**Site**				0.0
Beijing	77 (9.2)	73 (8.8)	150 (9.0)	
Helsingborg	10 (1.2)	10 (1.2)	20 (1.2)	
Skellefteå	9 (1.1)	7 (0.8)	16 (1.0)	
Kalmar	74 (8.8)	75 (9.0)	149 (8.9)	
Lund	283 (33.7)	272 (32.8)	555 (33.2)	
Uppsala	170 (20.2)	176 (21.2)	346 (20.7)	
Västerås	177 (21.0)	176 (21.2)	353 (21.1)	
Örebro	41 (4.9)	40 (4.8)	81 (4.9)	
**Smoking status**				3.5
Current smoker	116 (14.3)	108 (13.5)	224 (13.9)	
Ex-smoker	124 (15.3)	127 (15.9)	251 (15.6)	
Never smoked	572 (70.4)	564 (70.6)	1136 (70.5)	
**Alcohol use**				15.7
Drinking alcohol	506 (71.6)	483 (69.2)	989 (70.4)	
Stopped drinking	7 (1.0)	3 (0.4)	10 (0.7)	
Non-drinkers	194 (27.4)	212 (30.4)	406 (28.9)	
**No. of comorbidities**				0.0
0	650 (77.3)	639 (77.1)	1289 (77.2)	
1	165 (19.6)	160 (19.3)	325 (19.5)	
2+	26 (3.1)	30 (3.6)	56 (3.4)	
**No. of ongoing medications**				0.0
0	289 (34.4)	278 (33.5)	567 (34.0)	
1	246 (29.3)	253 (30.5)	499 (29.9)	
2	170 (20.2)	190 (22.9)	360 (21.6)	
3	96 (11.4)	75 (9.0)	171 (10.2)	
4+	40 (4.8)	33 (4.0)	73 (4.4)	
**ASA classification**				0.0
I	292 (34.7)	253 (30.5)	545 (32.6)	
II	455 (54.1)	500 (60.3)	955 (57.2)	
III	93 (11.1)	76 (9.2)	169 (10.1)	
IV	1 (0.1)	0 (0.0)	1 (0.1)	
**T stage**				0.0
T0	8 (1.0)	7 (0.8)	15 (0.9)	
T1	511 (60.8)	471 (56.8)	982 (58.8)	
T2	240 (28.5)	248 (29.9)	488 (29.2)	
T3	40 (4.8)	48 (5.8)	88 (5.3)	
T4	8 (1.0)	8 (1.0)	16 (1.0)	
TX	34 (4.0)	47 (5.7)	81 (4.9)	
**N stage**				0.0
N0	683 (81.2)	661 (79.7)	1344 (80.5)	
N1	118 (14.0)	106 (12.8)	224 (13.4)	
N2	4 (0.5)	7 (0.8)	11 (0.7)	
N3	2 (0.2)	8 (1.0)	10 (0.6)	
NX	34 (4.0)	47 (5.7)	81 (4.9)	
**M stage**				0.0
M0	807 (96.0)	785 (94.7)	1592 (95.3)	
MX	34 (4.0)	44 (5.3)	78 (4.7)	
**HER2 status**				26.1
0–1+	437 (69.9)	401 (65.8)	838 (67.9)	
2+	139 (22.2)	153 (25.1)	292 (23.7)	
3+	49 (7.8)	55 (9.0)	104 (8.4)	
**ER status**				14.6
Positive	629 (87.2)	633 (89.8)	1262 (88.5)	
Negative	92 (12.8)	72 (10.2)	164 (11.5)	
**PR status**				23.5
Positive	476 (74.0)	495 (78.1)	971 (76.0)	
Negative	167 (26.0)	139 (21.9)	306 (24.0)	
**Triple Negative Breast Cancer**				
Yes	41 (6.6)	26 (4.3)	67 (5.4)	26.1
**Neoadjuvant therapy**^a^				
Chemotherapy	61 (8.0)	58 (7.7)	119 (7.8)	9.0
Radiotherapy	1 (0.1)	7 (0.9)	8 (0.5)	9.0
Endocrine therapy	10 (1.3)	9 (1.2)	19 (1.2)	9.0
**Adjuvant therapy**				
Chemotherapy	251 (29.8)	252 (30.4)	503 (30.1)	0.0
Radiotherapy	576 (68.5)	564 (68.0)	1140 (68.3)	0.0
Endocrine therapy	572 (68.0)	572 (69.0)	1144 (68.5)	0.0

HER2 = Human epidermal growth factor receptor 2.

ER = Estrogen receptor.

PR = Progesterone recept.

a Only available for subjects diagnosed in Sweden.

**Table 2:** Anaesthetic technique and perioperative drugs in patients randomized to receiving either propofol or sevoflurane for anaesthesia maintenance during the index procedure and any anaesthesia within one year before and one year after the index operation.

	Propofol	Sevoflurane	Total	Missing
	n (%)	n (%)	n (%)	%
**Anaesthesia time–min, median [IQR]**	120.0 [100.0, 145.0]	125.0 [104.0, 152.0]	123.0 [102.0, 149.0]	0.0
**Anaesthesia with regional block/local**	531 (63.1)	518 (62.5)	1049 (62.8)	0.0
**Infiltration**				0.0
Paravertebral block	66 (7.8)	76 (9.2)	142 (8.5)	
Intercostal block	2 (0.2)	1 (0.1)	3 (0.2)	
Local infiltration	443 (52.7)	435 (52.5)	878 (52.6)	
**Anaesthesia, other**	51 (6.1)	57 (6.9)	108 (6.5)	0.0
**Opioids, usage at index surgery**				0.0
Oral opioids preop.	398 (47.3)	400 (48.3)	798 (47.8)	
Fentanyl	205 (24.4)	576 (69.5)	781 (46.8)	
Ketobemidone	143 (17.0)	130 (15.7)	273 (16.3)	
Morphine	123 (14.6)	122 (14.7)	245 (14.7)	
Remifentanil	726 (86.3)	349 (42.1)	1075 (64.4)	
Sufentanil	75 (8.9)	71 (8.6)	146 (8.7)	
Alfentanil	3 (0.4)	6 (0.7)	9 (0.5)	
**Opioids, dosage at index surgery**				0.0
Oral opioids preop.–mg; median [IQR]	5.0 [3.0–10.0]	6.0 [3.0–10.0]	5.3 [3.0–10.0]	
Fentanyl–μg; median [IQR]	100.0 [100.0–150.0]	100.0 [100.0–150.0]	100.0 [100.0–150.0]	
Ketobemidone–mg; median [IQR]	2.5 [2.5–5.0]	2.5 [2.5–3.0]	2.5 [2.5–5.0]	
Morphine–mg; median [IQR]	4.0 [2.0–5.0]	3.0 [2.0–5.0]	4.0 [2.0–5.0]	
Remifentanil–μg; median [IQR]	1032.0 [741.8–1450.0]	805.8 [617.0–1072.5]	952.0 [690.0–1304.0]	
Sufentanil–μg; median [IQR]	26.2 [20.0–41.7]	29.5 [21.6–40.1]	28.6 [20.4–40.6]	
Alfentanil–mg; median [IQR]	0.5 [0.5–1.3]	0.5 [0.5–0.5]	0.5 [0.5–0.5]	
**Opioid usage postoperatively**				0.0
Oral opioids postop.	189 (22.5)	162 (19.5)	351 (21.0)	
Alfentanil	1 (0.1)	0 (0.0)	1 (0.1)	
Fentanyl	0 (0.0)	9 (1.1)	9 (0.5)	
Ketobemidone	90 (10.7)	64 (7.7)	154 (9.2)	
Morphine	106 (12.6)	118 (14.2)	224 (13.4)	
Meperidine	1 (0.1)	0 (0.0)	1 (0.1)	
Remifentanil	7 (0.8)	1 (0.1)	8 (0.5)	

The five-year survival rate was 91.9% (95% CI 90.1–93.8) (773 out of 841) and 92.2% (90.3–94.0) (764/829), for the propofol and sevoflurane groups, respectively (HR = 1.03 (0.73–1.44); P = 0.875) in the ITT-analysis (Table 3). The corresponding outcome in the PP-analysis was 91.9% (90.0–93.8) (733/798) and 92.0% (90.0–94.0) (653/710) (HR = 1.01 (0.71–1.44); P = 0.955). HR, when post hoc adjusted for the uneven distribution of triple-negative cancer, was 1.01 (0.72–1.42), P = 0.944, and 1.03 (0.73–1.45), P = 0.870, when adjusted for repeated general anaesthesia (Table 3).

**Table 3:** Surgical procedures, blood loss, and complications in patients randomised to receive either propofol or sevoflurane for anaesthesia maintenance during the index procedure.

	Propofol	Sevoflurane	Total	Missing
	n (%)	n (%)	n (%)	%
**Type of surgery**^a^				14.3
Partial mastectomy	482 (66.5)	460 (65.2)	942 (65.8)	
Mastectomy	243 (33.5)	246 (34.8)	489 (34.2)	
**Surgery time–min, median [IQR]**	79.0 [60.0, 101.0]	84.0 [64.0, 108.0]	81.0 [62.0, 105.0]	0.0
**Blood loss–mL, median [IQR]**	20.0 [0.0, 50.0]	25.0 [0.0, 50.0]	20.0 [0.0, 50.00]	0.2
**Re-surgery within 30 days, no. of patients**	63 (7.5)	59 (7.1)	122 (7.3)	0.0
**Complications**				
Intraoperative complications	10 (1.2)	6 (0.7)	16 (1.0)	0.0
Surgical complications	188 (22.4)	197 (23.8)	385 (23.1)	0.0
**Medical complications**				
Thrombosis, lungs	2 (0.2)	0 (0.0)	2 (0.1)	0.0
Thrombosis, other	3 (0.4)	0 (0.0)	3 (0.2)	0.0
Thrombosis, peripheral (not in the brain)	2 (0.2)	0 (0.0)	2 (0.1)	0.0
Stroke	0 (0.0)	1 (0.1)	1 (0.1)	0.0
**Other complications**				0.0
None	797 (94.8)	787 (94.9)	1584 (94.9)	
1 complication	36 (4.3)	34 (4.1)	70 (4.2)	
2 complications	8 (1.0)	7 (0.8)	15 (0.9)	
3 complications	0 (0.0)	1 (0.1)	1 (0.1)	

a Only available for Swedish subjects.

In all, 183 patients died during the full observation period, 91 of 841 (10.8%) in the propofol group and 92 of 829 (11.1%) in the sevoflurane group. Of those who died, 11 did so within the first 365 days, and two of those within the first 30 days. Postoperatively, five patients with thromboses and two with pulmonary embolism were found in the propofol group and none in the other group (P = 0.062, Fisher's test) (Table 3). One of the patients with pulmonary embolism died within 30 days (no autopsy was performed; the clinical cause of death was pulmonary embolism). No major harm or unintended effects were otherwise reported. Medical and surgical complications are summarised in Table 4.

**Table 4:** Primary endpoint (5-year survival) in the primary study population and in different subgroups.

**Study Population**	Propofol	Sevoflurane	5 year survival (95% CI)							
	Deaths/Population	Deaths/Population	Propofol	Sevoflurane	HR (95% CI)	P-value	aHR (95% CI)^a^	P-value	aHR (95% CI)^b^	P-value
Intention-to-treat (ITT)	68/841	65/829	91.9 (90.1–93.8)	92.2 (90.3–94.0)	1.03 (0.73–1.44)	0.875	1.01 (0.72–1.42)	0.944	1.03 (0.73–1.45)	0.870
Per-protocol (PP)	65/798	57/710	91.9 (90.0–93.8)	92.0 (90.0–94.0)	1.01 (0.71–1.44)	0.955				
ITT with no re-anesthesia	54/667	52/631	91.9 (89.9–94.0)	91.8 (89.6–93.9)	0.98 (0.67–1.43)	0.902				
ITT with no complications	43/621	36/604	93.1 (91.1–95.1)	94.0 (92.2–95.9)	1.16 (0.75–1.81)	0.507				

a Adjusted Hazard Ratio–The model was adjusted for Triple Negative Breast Cancer (yes/no).

b Adjusted Hazard Ratio–The model was adjusted for re-anaesthesia within 30 days (yes/no), and between 31 and 365 days (yes/no), and complications (yes/no).

**Fig. 2:**
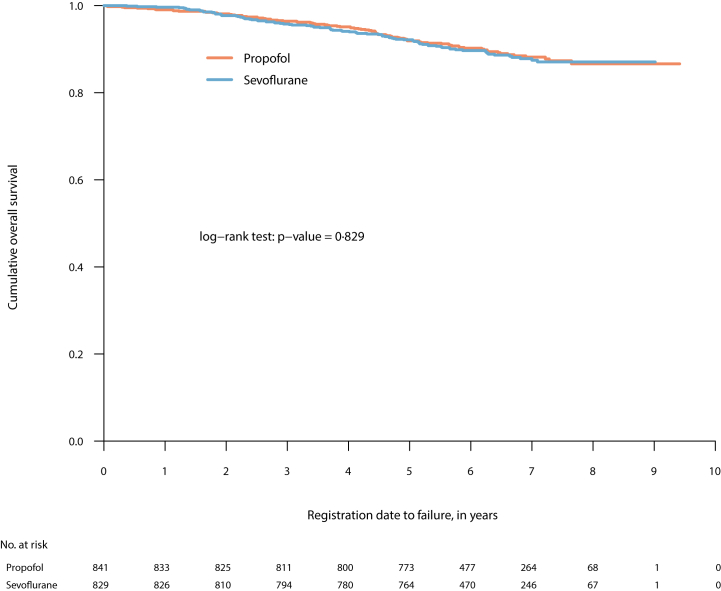
Kaplan-Meier curve for cumulative overall survival after propofol- or sevoflurane-based anaesthesia for breast cancer (Intention-to-Treat analysis).

Kaplan-Meier survival curves from the start of inclusion, almost nine years earlier to the end of follow-up, did not indicate any difference in survival between the groups (HR 0.97 (0.73–1.29); P = 0.829, log rank test; ITT) (Fig. 2) (HR 0.95 (0.70–1.28); P = 0.717, log rank test; PP) (Fig. S1). Data for cancer specific survival were available for the 1520 Swedish patients, of whom 85 died due to their cancer during the follow-up period, evenly distributed between the study groups (Fig. S2).

In pre-defined subgroup analyses, we could not find a site effect (data not shown), nor any impact of repeated general anaesthesia, predefined complications, or the use of paravertebral blocks (Figs. S3–S5). Increasing age, BMI, ASA classification, and number of co-morbidities, were statistically associated with the outcome in the univariable Cox regression analysis together with polypharmacy, smoking, no alcohol use, and more severe oncology characteristics (Table S1). Post hoc, we found that survival for triple-negative patients did not differ between study groups (P = 0.41, log-rank-test) (Table S1, Fig. S6).

## Discussion

In this RCT, we found no statistical evidence of a clinically relevant difference in 5-year survival between patients anaesthetised with either propofol or sevoflurane, when examining the possible effect of the choice of general anaesthesia for breast cancer surgery.

This finding contradicts some retrospective studies of other cancer types, where better survival has been found for cancer patients who were anaesthetised with propofol compared with those anaesthetised with sevoflurane.^2^ Our finding also contradicts breast cancer biomarker studies, in which propofol and inhaled anaesthetics seemed to affect hypoxia-inducible factor and the immune system in opposite directions, indicative of a more positive outcome for patients given propofol.^6^, ^7^, ^8^^,^^18^, ^19^ But the result is in line with an RCT on circulating cancer cells in breast cancer patients given either propofol or sevoflurane,^20^ observational studies on breast cancer specifically,^9^, ^10^, ^11^, ^12^, ^13^ and an RCT that examined the effect on cancer recurrence of a combination of propofol and a paravertebral block compared with sevoflurane and an opioid.^14^

A potential weakness of this study is that the protocol needed to be pragmatic, allowing contributing sites to vary all other aspects of anaesthesia beyond the choice of anaesthetic, such as other treatment interventions and surgical procedures. On the other hand, this circumstance may increase external validity and generalisability. A pragmatic decision not to record the dose of sevoflurane was made to simplify for the anaesthetic staff, and cerebral monitoring was not mandatory. These decisions make it impossible to determine whether the groups were anaesthetised to the same depth. However, we have no reason to believe that either group was systematically under- or overdosed. No data to date link anaesthetic dose to long-term survival. Another shortcoming in our study is that the autopsy frequency is low in Sweden, so the result for cancer-specific mortality may have some inherent uncertainty. It should also be noted that there are partially missing values for some variables.

As in several biomarker studies,^6^, ^7^, ^8^^,^^19^ propofol was used for pragmatic reasons for induction of general anaesthesia in the sevoflurane group. Since the given propofol could theoretically outweigh a negative effect of sevoflurane, this may be considered a potential protocol-caused effect modification. However, it has been observed that although increased doses of propofol lower 1-year mortality in patients without tumours, this is not the case for patients with a solid tumour such as breast cancer.^21^ This suggests that the use of propofol in the sevoflurane group would not have changed the outcome in the current study.

For the present study, the Hazard Ratio for overall survival was not adjusted for any potential confounders. This was due to an overall lack of imbalance between the groups and to mitigate a potential overfitting problem. An exception was the uneven distribution of triple negative cancers. This sub-type, with poor prognosis, was predominantly represented in the propofol group, 6.6% vs 4.3% (Table 1). The numbers are small, and a quarter of the HER-2 values are missing, but in a worst-case scenario of fast and poor outcome for all patients with triple negative cancer, a difference of 2.3 percentage-points between the study groups would represent a clinically meaningful difference. This was however not the case, although the Kaplan-Meier graph, with the low number of patients, suggests so (Table S1, Fig. S6). It would be far-fetched to speculate in an uneven distribution of any hitherto unknown confounder or effect modifier. Unlike RCTs with many exclusion criteria, resulting in a dominance of younger patients with less comorbidity, we had few exclusion criteria and no upper age limit. The external validity and generalisability should benefit from such circumstances.

The use of paravertebral blocks was not randomised in our study. The relatively few blocks that were applied, 8.6% of all patients, were utilised according to local practice, and evenly distributed between the study groups with no significant effect on overall survival (Table S1, Fig. S5). The use of fentanyl and remifentanil differed between the two groups. Remifentanil was used proportionally more with propofol, while fentanyl was used more in combination with sevoflurane. The median dose of remifentanil was higher in the propofol group. This difference probably had no unique effect on survival.

Synthetic opioids, such as fentanyl, alfentanil, and remifentanil, appear inert in respect of cancer pathology, whereas morphine has a suggested suppressive effect on the immune response.^22^, ^23^, ^24^, ^25^, ^26^, ^27^

This negative effect of morphine has been questioned recently, to the extent that it may even be considered protective against cancer recurrence and metastases.^28^^,^^29^ Inspired by such revelations, we determined post-hoc, that of the 183 deceased patients, 41.5% received postoperative opioids within the first 30 days postoperatively, compared with 36.3% in survivors (P = 0.17, Fisher's test). This finding, if statistically significant, could well be understood if opioids were given more frequently to more severely ill individuals, i.e., bias by indication. The observed, statistically non-significant overrepresentation of postoperative opioids among the deceased in the current study may support the former theory more than the latter. We suggest a larger study powered by the proportions found here.

Our analyses confirm that the most important predictors of breast cancer survival are patient related factors, such as co-morbidity and aggravating tumour characteristics (Table S1). Younger age, normal BMI, and non-smoking retain their positions as protective factors, although small. No alcohol use seemed to be a significant risk factor, which is not explained by less alcohol use by the elderly, with inherently shorter life expectancy (data not shown). As expected, mastectomy, which is performed for more extensive cancer, was associated with a less favourable outcome than partial mastectomy.

Obviously, different cancers may have varying properties to cause local recurrence or metastases, i.e., tumour biology differs, and the activation of adrenergic-inflammatory stimulus may differ as well.^30^ If anaesthetic drugs modify tumour biology, the resulting effect may vary between different cancers or different surgical procedures. Breast cancer surgery is a relatively short procedure with short exposure to the anaesthetic drug, and the superficial location of the cancer may make it easier to manage with a lower risk of cancer cell dissemination. Thus, there are reasons not to extrapolate the results of the current study to other cancer types.

To conclude, the present analysis of the randomised and controlled CAN study, comparing propofol- and sevoflurane-based general anaesthesia in 1670 breast cancer patients, did not demonstrate any difference in long-term overall survival following primary breast cancer surgery.

## Contributors

ME, AB, AEnd, and LB contributed to study design.

ME applied for central grants and PF for a local grant.

ME, JL, FW, D-X W, AEkn, RA, PF, and LH acted as local primary investigators, and ME was the coordinating investigator.

ME, AEnd, JL, FW, D-X W, AEkn, RA, PF, LH, and IÖ all recruited patients to the trial.

AB did the statistical analysis.

ME and AB have directly accessed and verified the underlying data reported.

ME wrote the first draft of the report with input from AB, AE, and LB.

All authors had full access to all the data in the trial and had final responsibility for the decision to submit for publication after participation in the review process.

## Data sharing statement

Anonymised individual patient data collected for this study can be made available on request via the corresponding author, considering possible legal restrictions (Swedish and EU; GDPR) and after presenting a sound proposal and ethics approval. A signed data access agreement is mandatory, including a description of the conditions for data release and the requirements for data transfer, storage, archiving, publication, and co-authorship. Also, study protocol, statistical analysis plan, and informed consent form may be asked for. The time frame for data sharing of breast cancer patients in the CAN-study is defined from six months after publication of the current manuscript up to three years thereafter.

## Declaration of interests

Dr M. Enlund has received consulting fees from Nimbelle AB, honoraria for lectures from Mälardalen University, and is on the board of the ENCORE trial.

Dr L. Bergkvist has received honoraria for arranging a course in cooperation with AstraZeneca.

Dr J. Lundberg served as Editor on the Editorial Board of Acta Anaesthesiologica Scandinavica until February 2023.

All other authors have nothing to declare.

## Appendix A Supplementary data

Table S1

Figure S1 Kaplan-Meier curve for cumulative overall survival after propofol- or sevoflurane-based anaesthesia for breast cancer (Per Protocol analysis).

Figure S2 Kaplan-Meier curve for cumulative cancer specific survival after propofol- or sevoflurane-based anaesthesia for Swedish breast cancer patients.

Figure S3 Kaplan-Meier curve for cumulative overall survival after propofol- or sevoflurane-based anaesthesia for breast cancer without repeated general anaesthesia.

Figure S4 Kaplan-Meier curve for cumulative overall survival after propofol- or sevoflurane-based anaesthesia for breast cancer with no complications.

Figure S5 Kaplan-Meier curve for cumulative overall survival after propofol- or sevoflurane-based anaesthesia for breast cancer depending on combined with or without paravertebral block.

Figure S6 Kaplan-Meier curve for cumulative overall survival after propofol- or sevoflurane-based anaesthesia in triple negative breast cancer patients.
