# Supplementary material for: Impact of general anaesthesia on breast cancer survival: a 5-year follow up of a pragmatic, randomised, controlled trial, the CAN-study, comparing propofol and sevoflurane
Source: eClinicalMedicine. 2023 Jun 9;60:102037. doi: 10.1016/j.eclinm.2023.102037 (PMC10276257; doi:10.1016/j.eclinm.2023.102037)
Supplement: Appendix 1 and 2 [file mmc1.docx]

**Appendix 1. Recorded variables**

The following variables were recorded into an electronic CRF:

Study specific variable

• Randomisation, i.e., allocation to either propofol or sevoflurane-group and

confirmation of hypnotic agent used during surgery

Demographic variables including habits and general health

• Age, Sex

• Place of residence, zip code (proxy for socio-economic status)

• Height, Weight

• Smoking (pack years; 1 pack year = 20 cigarettes/day for a year) and

Alcohol use (standard drinks; 1 standard drink = 12 g pure ethanol)

• ASA classification

• Co-morbidity

• Chronic medication

• Other anaesthetics one year prior to and one year following the index procedure (date, duration, type)

Anaesthesia related variables

• Duration of current anaesthesia

• Dose of intraoperative opioids during current anaesthesia

• Other adjuvant intraoperative treatment such as inotropic drugs

• Accumulated time with mean arterial pressure (MAP) below 65 mmHg or above 130 mmHg

• Accumulated fluid balance at the end of surgery

• Blood loss

• Transfusion (units of packed red blood cells, plasma, and platelets)

• Pre-and postoperative laboratory analyses only if taken for clinical reasons

• Additional local anaesthesia or regional blockade (type of block, local anaesthetic agent, and dosage)

• Use of Patent Blue V

• Dose of postoperative morphine or other opioids

• Antiemetics and anti-inflammatory drugs

• Re-anaesthesia (type of)

Surgical variables

• Cancer location

• Date of surgery

• Duration of surgery

• Surgical (intraoperative) complications

• Postoperative complications / postoperative morbidity related to surgery

• Re-surgery during the first postoperative year (date, duration, and indication)

**Appendix 2. Participating study sites and number of included patients, respectively**

Beijing 200

Helsingborg 20

Kalmar 158

Lund 575

Skellefteå 16

Uppsala 357

Västerås 354

Örebro 84
