# Supplementary material for: Impact of general anaesthesia on breast cancer survival: a 5-year follow up of a pragmatic, randomised, controlled trial, the CAN-study, comparing propofol and sevoflurane
Source: eClinicalMedicine. 2023 Jun 9;60:102037. doi: 10.1016/j.eclinm.2023.102037 (PMC10276257; doi:10.1016/j.eclinm.2023.102037)
Supplement: Table S1 [file mmc2.docx]

Table S1. Relative risk of overall mortality expressed as Hazard Ratios (HR) and 95% confidence intervals (CI) using univariable Cox regression by study populations.

|  |  |  |  |  |  |
| --- | --- | --- | --- | --- | --- |
| **Exposure variable** | **Events** | **Population** | **HR** | **95% CI** | **p-value** |
| **Randomisation (ITT)** |  |  |  |  |  |
| Sevoflurane | 91 | 841 | 1·00 | reference |  |
| Propofol | 92 | 829 | 0·97 | 0·73-1·30 | 0·831 |
| **Randomisation (PP)** |  |  |  |  |  |
| Sevoflurane | 81 | 710 | 1·00 | reference |  |
| Propofol | 87 | 798 | 0·95 | 0·70-1·28 | 0·715 |
| **Age at randomisation** | 183 | 1670 | 1·01 | 1·01-1·02 | <0·001 |
| **BMI at randomisation** | 180 | 1643 | 1·01 | 1·01-1·02 | <0·001 |
| **Smoking status** |  |  |  |  |  |
| Current smoker | 44 | 224 | 1·00 | reference |  |
| Ex-smoker | 30 | 251 | 0·59 | 0·37-0·95 | 0·029 |
| Never smoked | 106 | 1136 | 0·48 | 0·34-0·68 | <0·001 |
| **Alcohol use*** |  |  |  |  |  |
| Drinking alcohol | 102 | 989 | 1·00 | reference |  |
| Non-drinkers | 51 | 406 | 1·42 | 1·01-1·99 | 0·043 |
| **No· of comorbidities** |  |  |  |  |  |
| 0 | 113 | 1289 | 1·00 | reference |  |
| 1 | 59 | 325 | 2·16 | 1·58-2·96 | <0·001 |
| 2+ | 11 | 56 | 2·26 | 1·22-4·19 | 0·010 |
| **No· of ongoing medications** |  |  |  |  |  |
| 0 | 40 | 567 | 1·00 | reference |  |
| 1 | 53 | 499 | 1·50 | 0·99-2·26 | 0·053 |
| 2 | 44 | 360 | 1·73 | 1·13-2·65 | 0·012 |
| 3 | 26 | 171 | 2·14 | 1·31-3·51 | 0·002 |
| 4+ | 20 | 73 | 4·12 | 2·41-7·05 | <0·001 |
| **ASA classification** |  |  |  |  |  |
| I | 22 | 545 | 1·00 | reference |  |
| II | 114 | 955 | 3·03 | 1·92-4·74 | <0·001 |
| III-IV | 47 | 170 | 7·59 | 4·57-12·59 | <0·001 |
| **Paravertebral block** |  |  |  |  |  |
| No | 167 | 1528 | 1·00 | reference |  |
| Yes | 16 | 142 | 1·07 | 0·64-1·79 | 0·786 |
| **T stage** |  |  |  |  |  |
| T0/TX | 15 | 96 | 1·00 | reference |  |
| T1 | 73 | 982 | 0·49 | 0·28-0·85 | 0·011 |
| T2 | 74 | 488 | 1·05 | 0·60-1·83 | 0·869 |
| T3-T4 | 21 | 104 | 1·45 | 0·75-2·82 | 0·270 |
| **N stage** |  |  |  |  |  |
| N0/NX | 142 | 1425 | 1·00 | reference |  |
| N1 | 38 | 224 | 1·86 | 1·30-2·67 | <0·001 |
| N2-N3 | 3 | 21 | 1·76 | 0·56-5·51 | 0·335 |
| **M stage** |  |  |  |  |  |
| M0 | 170 | 1592 | 1·00 | reference |  |
| MX | 13 | 78 | 1·49 | 0·85-2·62 | 0·165 |
| **HER2 status** |  |  |  |  |  |
| 0-1+ | 68 | 838 | 1·00 | reference |  |
| 2+ | 35 | 292 | 1·59 | 1·06-2·38 | 0·027 |
| 3+ | 9 | 104 | 1·11 | 0·56-2·23 | 0·231 |
| **ER status** |  |  |  |  |  |
| Positive | 116 | 1262 | 1·00 | reference |  |
| Negative | 25 | 164 | 1·80 | 1·17-2·78 | 0·008 |
| **PR status** |  |  |  |  |  |
| Positive | 83 | 971 | 1·00 | reference |  |
| Negative | 51 | 306 | 2·06 | 1·45-2·92 | <0·001 |
| **Triple Negative Breast Cancer** |  |  |  |  |  |
| No | 101 | 1167 | 1·00 | reference |  |
| Yes | 11 | 67 | 1·98 | 1·06-3·69 | 0·032 |
| **Type of surgery** |  |  |  |  |  |
| Partial mastectomy | 81 | 942 | 1·00 | reference |  |
| Mastectomy | 77 | 489 | 1·93 | 1·41-2·63 | <0·001 |
|  |  |  |  |  |  |
| * Subjects with stopped drinking were excluded (n=10) | |  |  |  |  |

ITT = Intention-To-Treat population

PP = Per Protocol population

HER2 = Human epithelial growth factor receptor 2

ER = Estrogen receptor

PR = Progesterone receptor
