# Supplementary figures and images for: Impact of general anaesthesia on breast cancer survival: a 5-year follow up of a pragmatic, randomised, controlled trial, the CAN-study, comparing propofol and sevoflurane
Source: eClinicalMedicine. 2023 Jun 9;60:102037. doi: 10.1016/j.eclinm.2023.102037 (PMC10276257; doi:10.1016/j.eclinm.2023.102037)

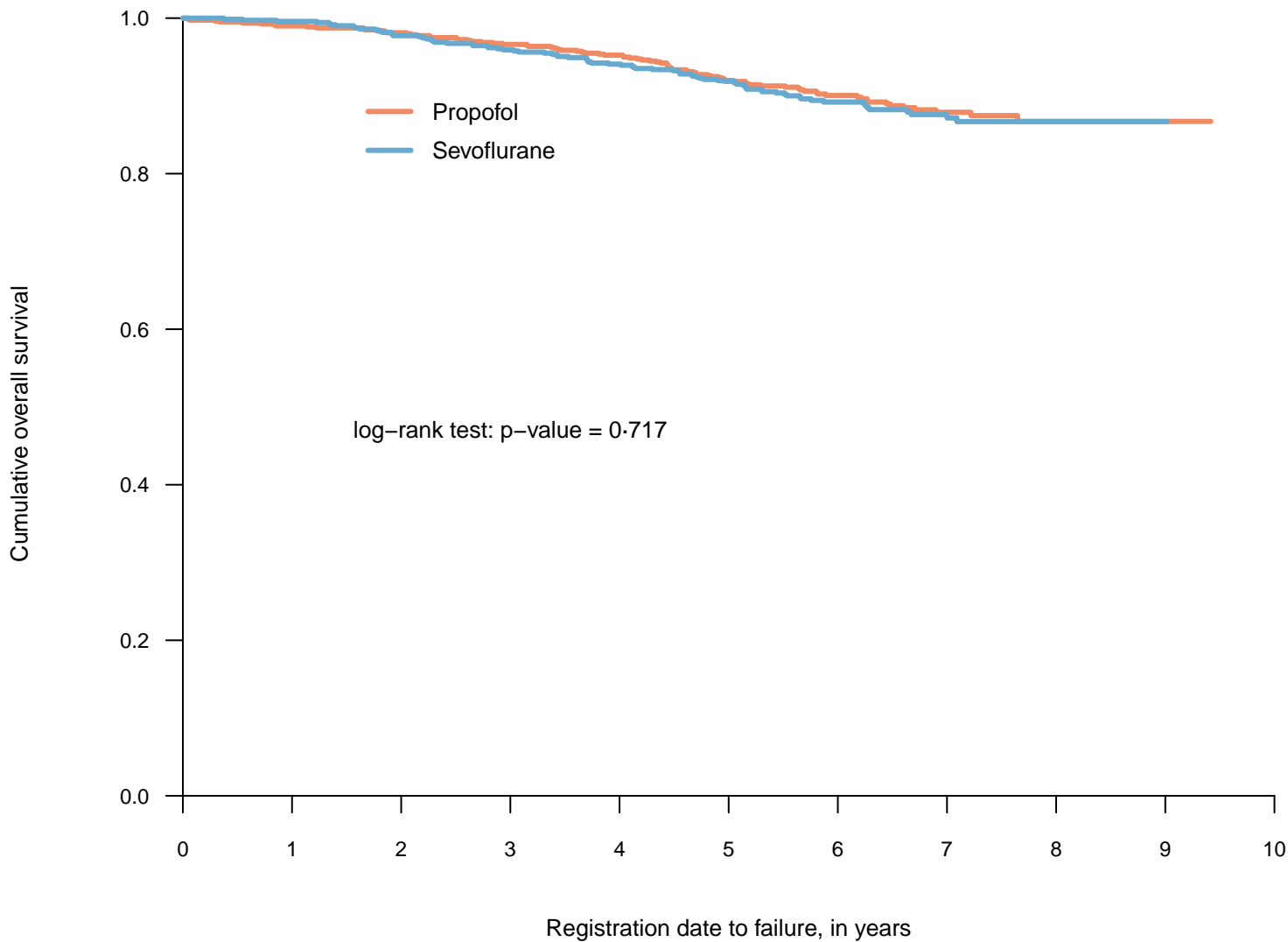

No. at risk

|             |     |     |     |     |     |     |     |     |    |   |   |
|-------------|-----|-----|-----|-----|-----|-----|-----|-----|----|---|---|
| Propofol    | 798 | 790 | 783 | 771 | 760 | 733 | 448 | 250 | 65 | 1 | 0 |
| Sevoflurane | 710 | 707 | 694 | 681 | 668 | 653 | 397 | 202 | 53 | 1 | 0 |

Supplement: Figure S1 — Kaplan-Meier curve for cumulative overall survival after propofol- or sevoflurane-based anaesthesia for breast cancer (Per Protocol analysis). [file mmc3.pdf]

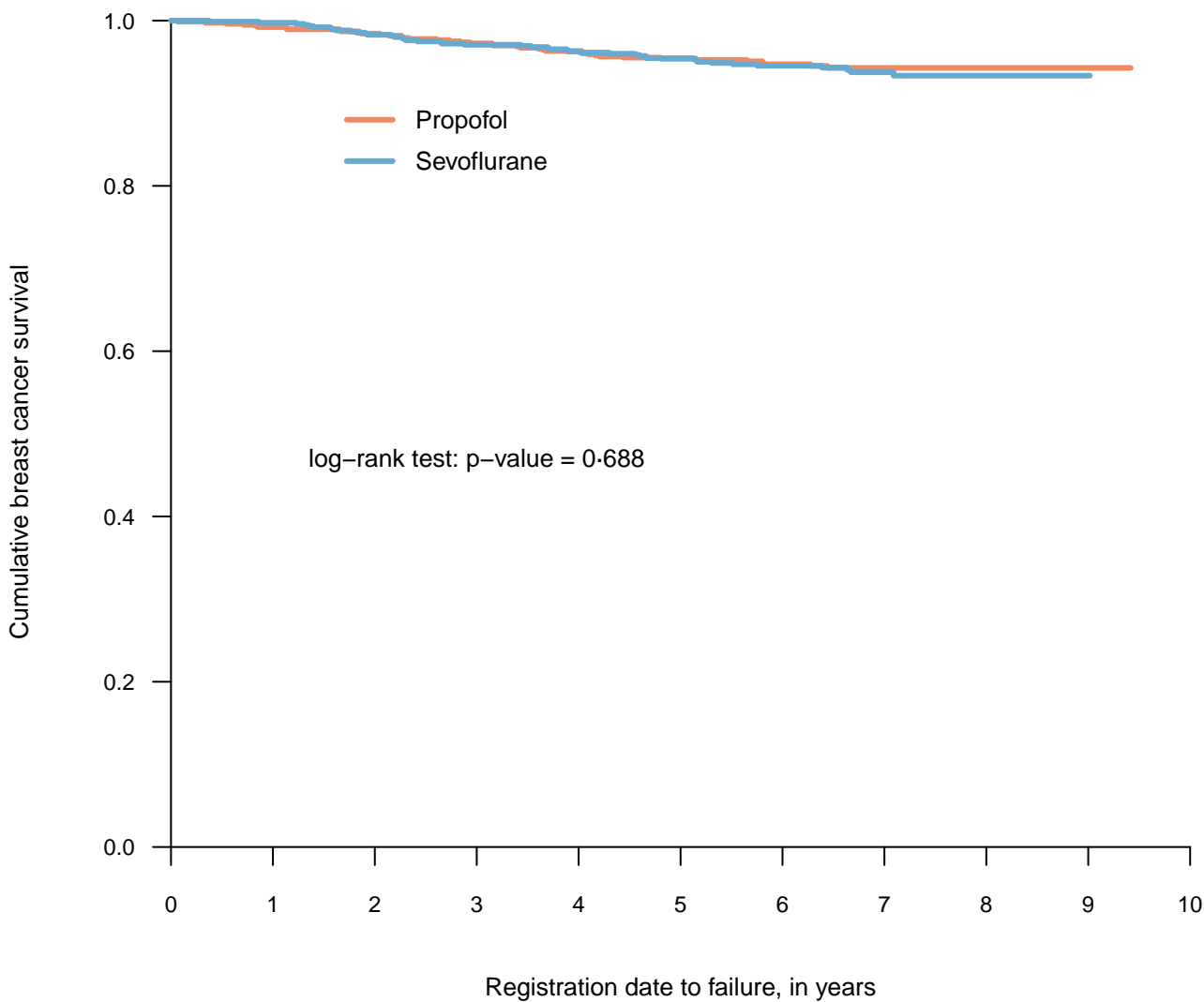

No. at risk

|             |     |     |     |     |     |     |     |     |    |   |   |
|-------------|-----|-----|-----|-----|-----|-----|-----|-----|----|---|---|
| Propofol    | 764 | 757 | 750 | 736 | 725 | 699 | 477 | 264 | 68 | 1 | 0 |
| Sevoflurane | 756 | 753 | 737 | 722 | 709 | 695 | 470 | 246 | 67 | 1 | 0 |

Supplement: Figure S2 — Kaplan-Meier curve for cumulative cancer specific survival after propofol- or sevoflurane-based anaesthesia for Swedish breast cancer patients. [file mmc4.pdf]

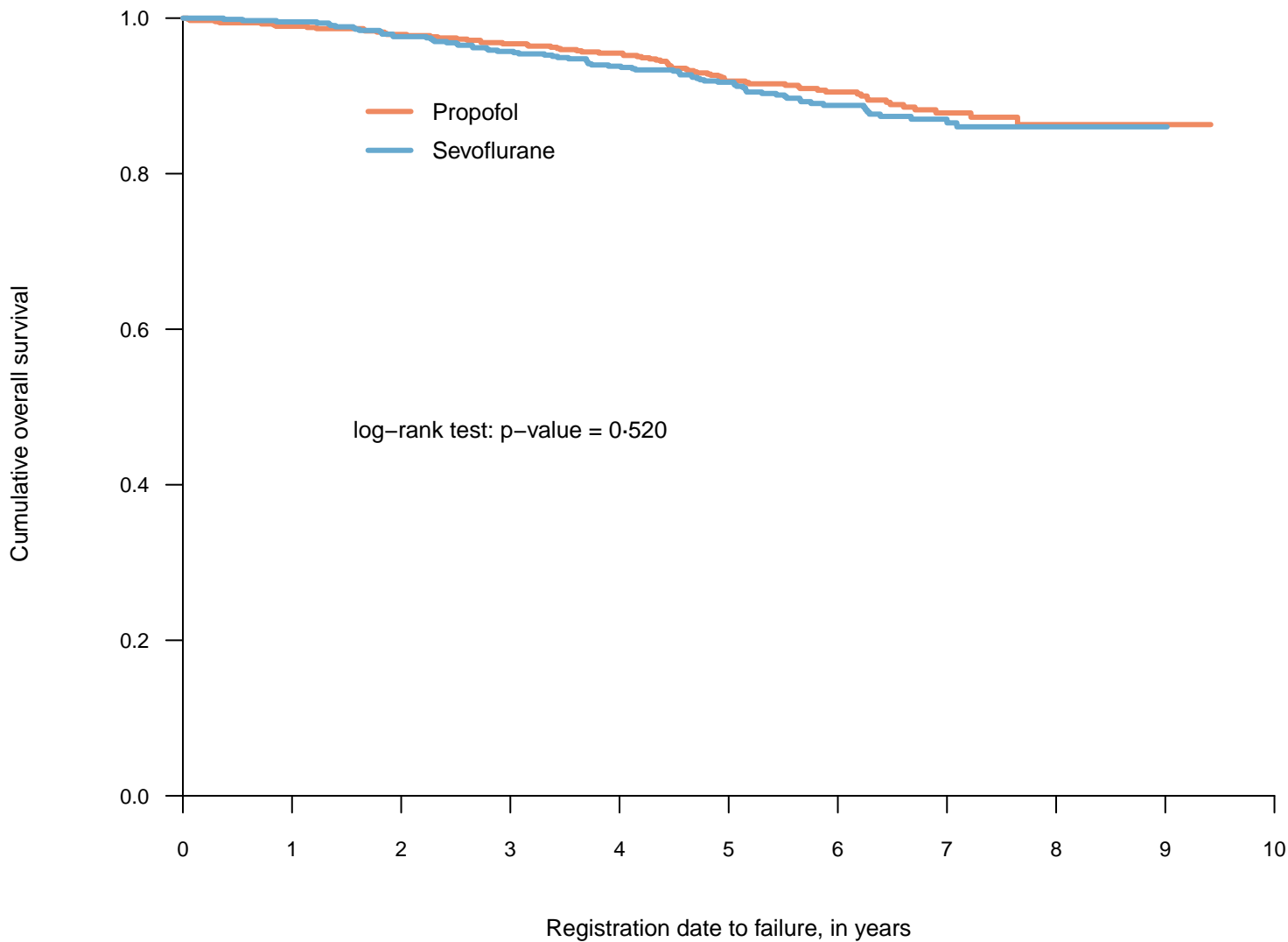

No. at risk

|             |     |     |     |     |     |     |     |     |    |   |   |
|-------------|-----|-----|-----|-----|-----|-----|-----|-----|----|---|---|
| Propofol    | 667 | 660 | 653 | 645 | 637 | 613 | 367 | 193 | 53 | 1 | 0 |
| Sevoflurane | 631 | 628 | 616 | 604 | 592 | 579 | 344 | 184 | 48 | 1 | 0 |

Supplement: Figure S3 — Kaplan-Meier curve for cumulative overall survival after propofol- or sevoflurane-based anaesthesia for breast cancer without repeated general anaesthesia. [file mmc5.pdf]

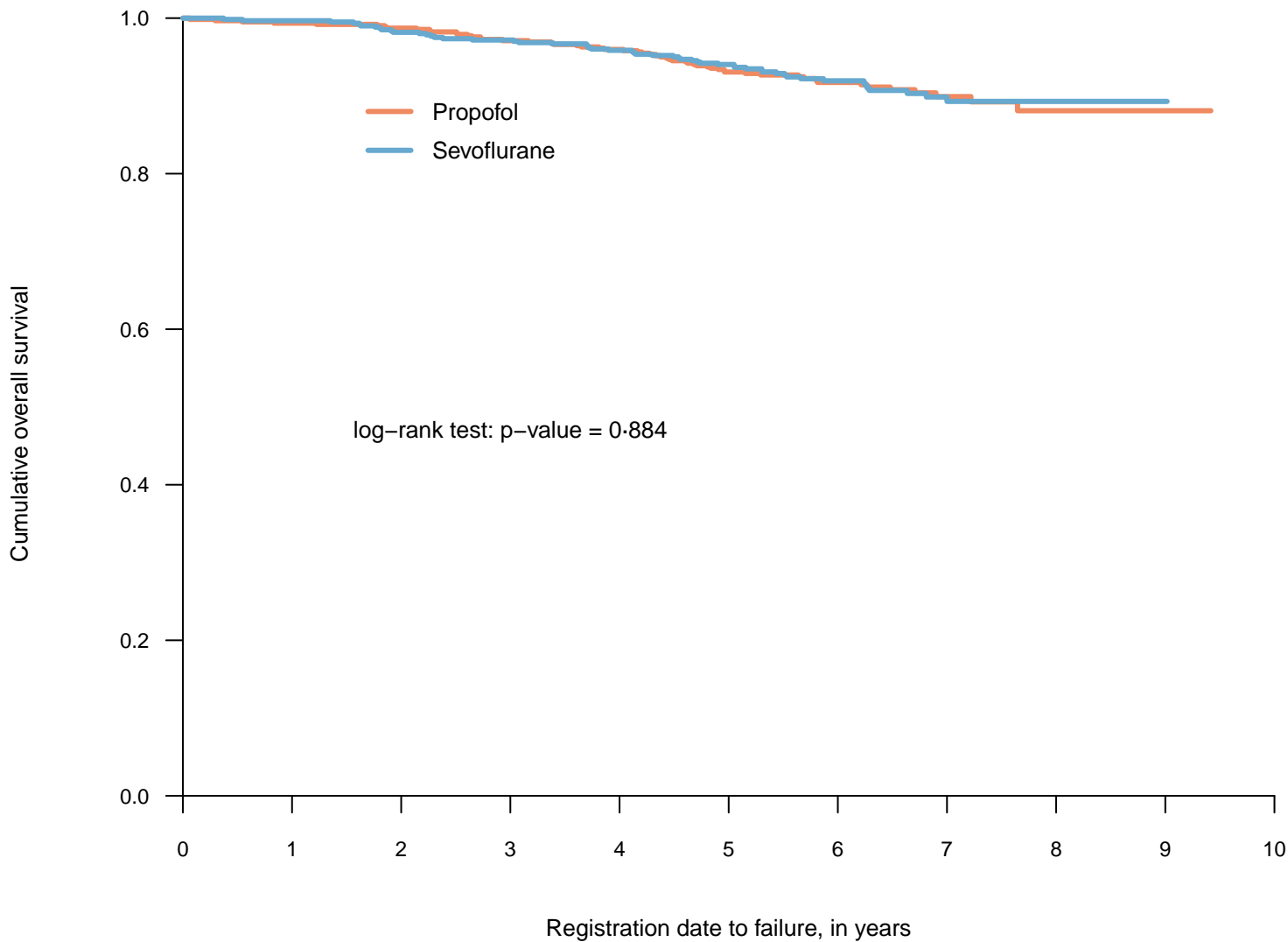

No. at risk

|             |     |     |     |     |     |     |     |     |    |   |   |
|-------------|-----|-----|-----|-----|-----|-----|-----|-----|----|---|---|
| Propofol    | 621 | 617 | 613 | 603 | 596 | 578 | 328 | 165 | 42 | 1 | 0 |
| Sevoflurane | 604 | 602 | 593 | 587 | 579 | 568 | 322 | 158 | 47 | 1 | 0 |

Supplement: Figure S4 — Kaplan-Meier curve for cumulative overall survival after propofol- or sevoflurane-based anaesthesia for breast cancer with no complications. [file mmc6.pdf]

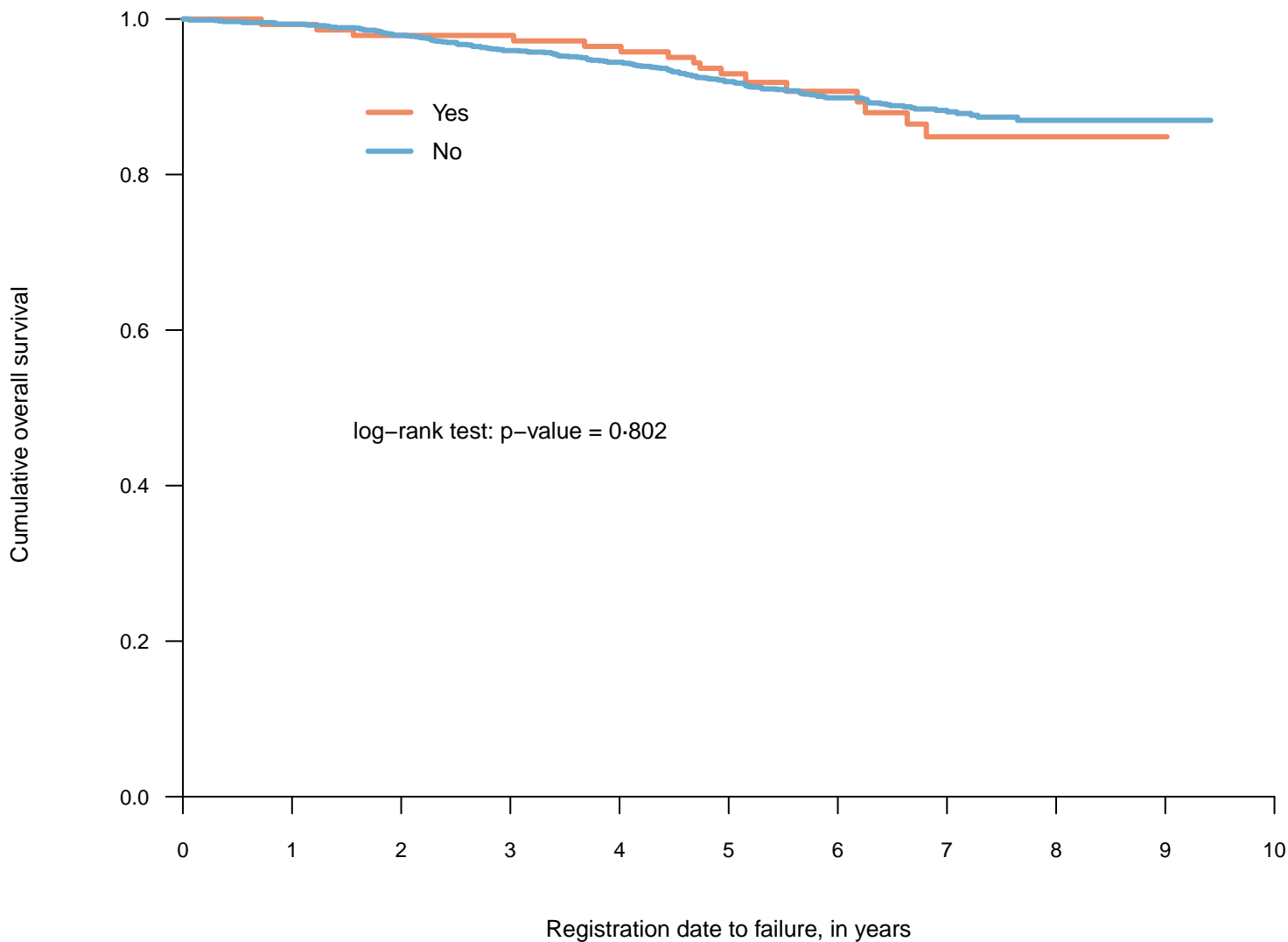

No. at risk

|     |      |      |      |      |      |      |     |     |     |   |   |
|-----|------|------|------|------|------|------|-----|-----|-----|---|---|
| Yes | 142  | 141  | 139  | 139  | 137  | 132  | 68  | 46  | 15  | 1 | 0 |
| No  | 1528 | 1518 | 1496 | 1466 | 1443 | 1405 | 879 | 464 | 120 | 1 | 0 |

Supplement: Figure S5 — Kaplan-Meier curve for cumulative overall survival after propofol- or sevoflurane-based anaesthesia for breast cancer depending on combined with or without paravertebral block. [file mmc7.pdf]

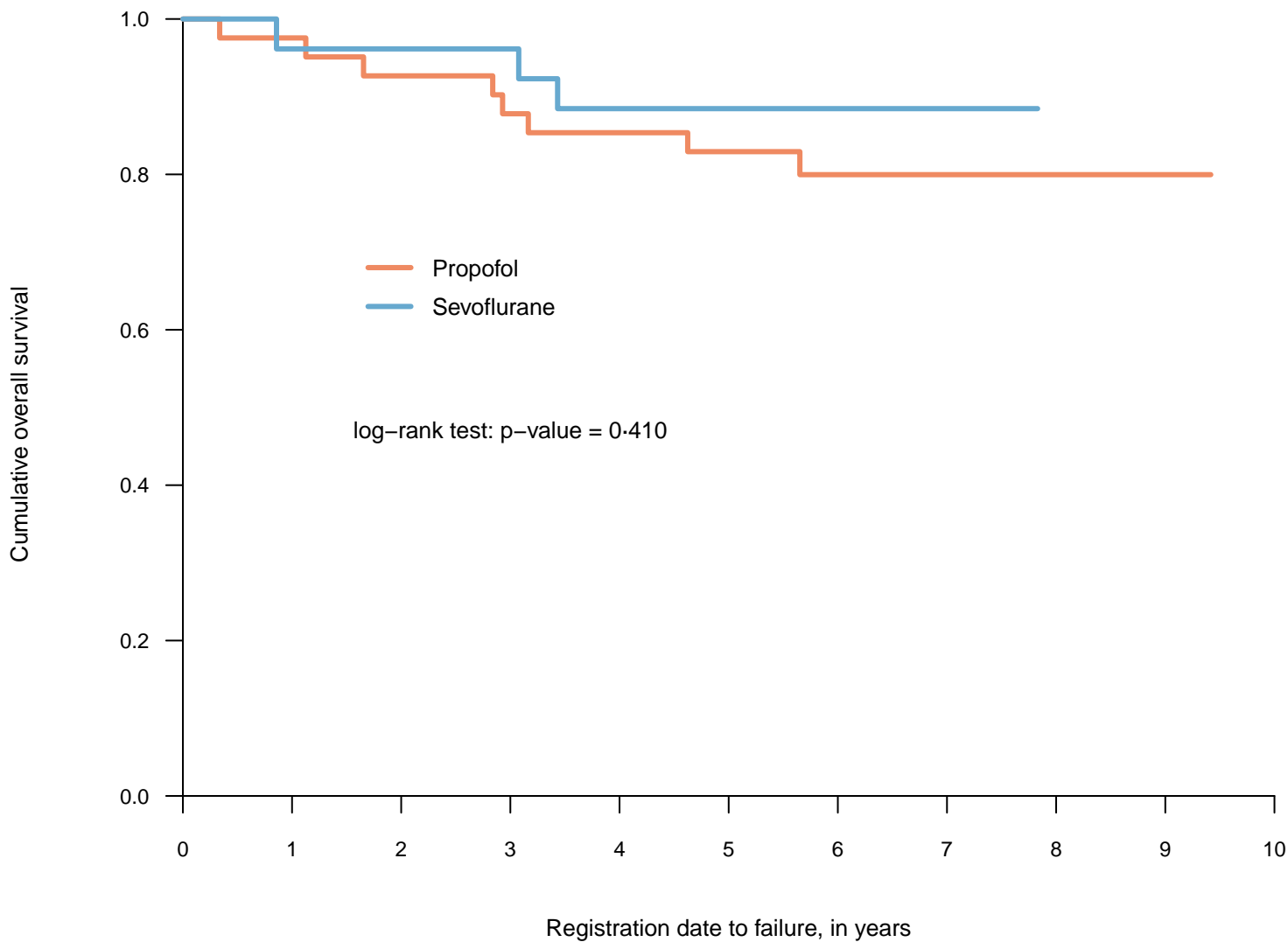

No. at risk

|             |    |    |    |    |    |    |    |   |   |   |   |
|-------------|----|----|----|----|----|----|----|---|---|---|---|
| Propofol    | 41 | 40 | 38 | 36 | 35 | 34 | 19 | 7 | 1 | 1 | 0 |
| Sevoflurane | 26 | 25 | 25 | 25 | 23 | 23 | 15 | 8 | 0 |   |   |

Supplement: Figure S6 — Kaplan-Meier curve for cumulative overall survival after propofol- or sevoflurane-based anaesthesia in triple negative breast cancer patients. [file mmc8.pdf]
